# Environmental Factors Shaping the Community Structure of Ammonia-Oxidizing Bacteria and Archaea in Sugarcane Field Soil

**DOI:** 10.1264/jsme2.ME14137

**Published:** 2014-12-27

**Authors:** Kanako Tago, Takashi Okubo, Yumi Shimomura, Yoshitomo Kikuchi, Tomoyuki Hori, Atsushi Nagayama, Masahito Hayatsu

**Affiliations:** 1 Environmental Biofunction Division, National Institute for Agro-Environmental Sciences3–1–3 Kannondai, Tsukuba, Ibaraki 305–8604Japan; 2 Bioproduction Research Institute, National Institute of Advanced Industrial Science and Technology (AIST) Hokkaido2–17–2–1 Tsukisamu-higashi, Toyohira-ku, Sapporo, Hokkaido 062–8517Japan; 3 Research Institute for Environmental Management Technology, AISTTsukuba, Ibaraki 305–8569Japan; 4 Okinawa Prefectural Agricultural Research CenterItoman, Okinawa 901–0336Japan

**Keywords:** ammonia-oxidizing archaea, ammonia-oxidizing bacteria, nitrification, pyrosequencing, soil pH

## Abstract

The effects of environmental factors such as pH and nutrient content on the ecology of ammonia-oxidizing bacteria (AOB) and archaea (AOA) in soil has been extensively studied using experimental fields. However, how these environmental factors intricately influence the community structure of AOB and AOA in soil from farmers’ fields is unclear. In the present study, the abundance and diversity of AOB and AOA in soils collected from farmers’ sugarcane fields were investigated using quantitative PCR and barcoded pyrosequencing targeting the ammonia monooxygenase alpha subunit (*amoA*) gene. The abundances of AOB and AOA *amoA* genes were estimated to be in the range of 1.8 × 10^5^–9.2 × 10^6^ and 1.7 × 10^6^–5.3 × 10^7^ gene copies g dry soil^−1^, respectively. The abundance of both AOB and AOA positively correlated with the potential nitrification rate. The dominant sequence reads of AOB and AOA were placed in *Nitrosospira*-related and *Nitrososphaera*-related clusters in all soils, respectively, which varied at the level of their sub-clusters in each soil. The relationship between these ammonia-oxidizing community structures and soil pH was shown to be significant by the Mantel test. The relative abundances of the OTU1 of *Nitrosospira* cluster 3 and *Nitrososphaera* subcluster 7.1 negatively correlated with soil pH. These results indicated that soil pH was the most important factor shaping the AOB and AOA community structures, and that certain subclusters of AOB and AOA adapted to and dominated the acidic soil of agricultural sugarcane fields.

Nitrification, the microbial oxidation of ammonia to nitrate, is a key process in the global nitrogen cycle (13). In agricultural soil, nitrification is directly involved in plant nitrogen nutrition and soil nitrogen losses through the leaching of nitrate, and is coupled with denitrification, which leads to water pollution and contributes to the production of the greenhouse gas nitrous oxide (8, 33, 35, 36, 42). Microbial ammonia oxidation is the first and rate-limiting step in the process of nitrification. Therefore, ammonia-oxidizing microorganisms have received considerable attention from both agricultural and environmental viewpoints.

The oxidation of ammonia is mediated by ammonia-oxidizing bacteria (AOB) and ammonia-oxidizing archaea (AOA) (32). To understand the relative importance of these different groups in agricultural soils, many studies have investigated the effects of soil chemical and physical properties as well as agricultural management strategies such as fertilizer, water control, and crop type on the diversity, abundance, and activity of AOB and AOA (39). These studies revealed that soil pH was a major factor affecting AOB and AOA community structures and their activities (9, 29). A number of molecular ecological studies recently demonstrated that the abundance of AOA was several orders higher than that of AOB in various acidic soils (48, 49). In addition to pH, the kind and quantity of nitrogen fertilizer have been shown to have a significant impact on these ammonia-oxidizing communities (14). AOB abundance was increased by the addition of nitrogen fertilizer and positively correlated with potential nitrification activity in soils supplied with large amounts of fertilizer (19, 47), whereas AOA growth was suppressed by high levels of ammonium in soil microcosms (46). Most of these findings were obtained in studies using microcosms and experimental fields that were stringently controlled for scientific purposes.

In the soil of agricultural (farmers’) fields, various environmental factors, such as soil pH and inorganic nitrogen content, can collectively influence the community structures of AOB and AOA (20). Therefore, to understand the role of AOB and AOA in transforming nitrogen nutrients in agricultural soils, the ecology of AOB and AOA must be studied in a wide variety of agricultural fields. From a practical standpoint, these studies would ideally lead to the development of nitrification control technologies to prevent nitrogen loss from and the pollution of agricultural fields. However, only a limited number of studies have been conducted using farming-scale agricultural fields; therefore, it currently remains unclear whether AOB or AOA are the main contributors to nitrification, and how environmental factors intricately shape the community structures of AOB and AOA in agricultural fields remains to be determined.

The aim of the present study was to assess the activity, abundance, and diversity of AOB and AOA in agricultural sugarcane fields with similar soil types and under the same climate conditions. We here reported the abundance and diversity of AOB and AOA analyzed by *amoA* gene-based quantitative polymerase chain reaction (qPCR) and pyrosequencing, and the results of statistical analyses performed to evaluate the relationship between the community structures of ammonia oxidizers and soil properties.

## Materials and Methods

### Soil samples

As a first step towards understanding the ecology of AOB and AOA in agricultural (farmers’) field soil, we selected sugarcane fields located on Minami-Daito Island to exclude the effects of climate conditions, crop types, and soil types on AOB and AOA, which would have complicated the analysis and interpretation of the results. In Minami-Daito Island, sugarcane is generally planted in spring and summer and harvested in winter and the following winter, respectively. Nitrogen fertilizer is generally applied three times, involving a basal dressing and two additional dressings. Soil samples were obtained from thirteen agricultural sugarcane fields in Minami-Daito Island located in the Philippine Sea (25°50′N, 131°14′E), 360 km east of Okinawa Island, Japan (Fig. S1). Sugarcane fields cover approximately 60% of the total island area (30.57 km^2^). The climate of the island is temperate, with a mean annual temperature of 24°C and mean annual precipitation of 1,000 mm. The soil types were lateritic red and lateritic yellow (25). Three separate soil subsamples were taken from each sugarcane field at a depth of 1–10 cm with a small sterile shovel and sieved through a 2-mm pore size mesh. The surface soil at a depth of 0 to 1 cm was removed because the soil water content was extremely low. The three subsamples were separately used in a soil analysis and DNA extraction. The samples were stored at −80°C until DNA analysis and the remaining soil was stored at 4°C until analysis of nitrification potential (less than 7 d).

### Soil analysis

To measure the contents of NO_3_^−^ and NH_4_^+^, soil samples (10 g) were extracted with 100 mL of 2 M KCl, and the suspension was passed through Whatman no. 10 filter paper. Nitrate was analyzed using the copper-cadmium reduction method, while NH_4_^+^ was analyzed by the indophenol blue method in a continuous flow analyzer (TRRACS, Bran + Luebbe, Norderstedt, Germany). Soil pH was determined in soil/water suspensions (1/2.5 w/v). Total nitrogen and carbon contents were determined by an elemental analyzer (2400II CHNS/O, PerkinElmer, USA). Available phosphate was extracted from soils with 2 mM sulfuric acid (1:200 soil: solution ratio) for 30 min and the phosphate in the extract was measured calorimetrically using the molybdenum blue method.

### Potential nitrification rate

The potential nitrification rate (PNR) was determined as the rate of NO_2_^−^ accumulation through the inhibition of NO_2_^−^ oxidation with chlorate according to the method of Belser and Mays (3). Briefly, soil samples (2.5 g) were mixed with 10 mL of phosphate buffer (pH 7.0) containing 1 mM (NH_4_ )_2_ SO_4_ and 10 mM KClO_3_ . Soil slurries in a 50-mL plastic tube were incubated for several h at 25°C with shaking at 120 rpm. One-milliliter aliquots of slurry were collected every h over a period of 1 to 4 h of incubation, and centrifuged at 10,000×*g* for 10 min. The concentration of accumulated NO_2_^−^ in the supernatant was spectrophotometrically determined using the Griess-Ilosvay method. Nitrification rates were calculated based on the accumulation of nitrite (nmole) h^−1^ g of dry soil^−1^.

### DNA extraction from soil

Soil DNA was extracted from each of the three subsamples (0.4 g) using the Fast DNA Spin Kit for soil (Qbiogene, Inc., Irvine, CA, USA) according to the manufacturer’s instructions. Cell lysis was performed by vigorous shaking in a bead beater at an intensity of 5.5 for 30 s (FastPrep DNA Extractor, Qbiogene, Inc., Irvine, CA, USA). DNA was finally eluted with 80 μL of the DNA elution solution included in the kit. The extracted soil DNA was then purified using a DNA Clean & Concentrator-25 DNA Purification Kit (Zymo Research, Irvine, CA, USA), and stored at −80°C until later analysis.

### Quantitative PCR for *amoA*

The AOB and AOA *amoA* gene copies were quantified by a SYBR Green I-based qPCR technique using a Step OnePlus real-time PCR system (Applied Biosystems, Foster City, CA, USA) with SYBR Premix EX Taq (TaKaRa Bio Inc., Shiga, Japan). The primer pair amoA1F (34) and amoA2IR (2) was used to quantify the AOB *amoA* gene. The 20-μL reaction volume contained 10 μL SYBR Premix Ex Taq (TaKaRa Bio Inc.), 2 μg of bovine serum albumin, 400 nM of each AOB *amoA* gene primer, and 2 μL of 10-fold diluted or undiluted extracted DNA as a template. Three replicates were analyzed for each sample. Amplifications were carried out under the following cycle conditions: 94°C for 2 min, followed by 40 cycles of 30 s at 94°C, 30 s at 56°C, and 30 s at 72°C. The primer pair amoA19IF and amoA 643IR (28) was used to quantify the AOA *amoA* gene. The cycle conditions were the same as those for the AOB *amoA* gene, except for the primer concentration (200 nM) and annealing temperature (54°C). The AOA *amoA* gene fragment (GenBank/EMBL/DDBJ accession number AB569307) and AOB *amoA* gene fragment from *Nitrosospira multiformis* ATCC25196 (GenBank/EMBL/DDBJ accession number U91603), subcloned into the pGEM-T Easy vector (Promega, Madison, WI, USA), were used to generate a standard curve.

### Pyrosequencing analysis of *amoA*

Eight out of thirteen soil samples were used in the 454-pyrosequencing analysis. The remaining five samples were excluded from the analysis due to the insufficient yields of their PCR products. Fusion primers for pyrosequencing containing specific sequences (*amoA* gene, beads, or sequence primer attachment site, key, and MID sequences) were used for PCR amplification. PCR amplification was conducted in triplicate under the same conditions as those for the qPCR analysis. The amplified DNA fragments were purified by a QIAquick PCR Purification Kit (QIAGEN, Valencia, CA, USA). The purified fragments were subjected to agarose gel electrophoreses, and the target DNA fragments in the gel were purified using the QIAquick Gel Extraction Kit (QIAGEN). The quality of the purified DNA fragments was assessed using a Bioanalyzer (Agilent Technologies, Santa Clara, USA). The quantities of the DNA fragments were determined by the Quant-iT PicoGreen dsDNA Assay Kit (Life Technologies, Carlsbad, CA, USA). Emulsion PCR and pyrosequencing were performed using the Roche 454 Junior sequencer (Roche, Nutley, NJ, USA) according to the 454 Life Sciences protocol (Roche Diagnostics, Branford, CT, USA).

### Sequence analysis and phylogenetic assignment

Raw sequences obtained by pyrosequencing were screened by eliminating sequences with a low quality (quality score < 25) and of insufficient length (AOB *amoA* < 450 nt and AOA *amoA* < 425 nt). Unique sequences among the trimmed sequences were obtained using the unique.seqs-function of the Mothur software (37). AOB *amoA* sequences longer than 450 nt and AOA *amoA* sequences longer than 598 nt were downloaded from the NCBI database. Unique sequences from the downloaded sequence files were obtained using Mothur as described above. These sequences were used as references when a chimera check of the pyrosequencing data was performed using the chimera.uchime-function in Mothur. The non-chimeric sequences were aligned using the dpparttree algorithm in the Mafft software (http://mafft.cbrc.jp/alignment/software/tips.html). A distance matrix of the aligned sequences was calculated using the dist.seqs-function in Mothur. Clustering of the sequence data was carried out using cluster-function with the furthest neighbor algorithm in Mothur. Representative sequences of each cluster were obtained using the get.oturep-function in Mothur. The phylogenetic analysis was performed using MEGA5 software (44).

### Statistical analysis

Pairwise correlations between soil properties and the abundance or relative abundance of AOB and AOA were performed by Pearson’s correlation coefficient using SPSS (IBM, New York, NY, USA). Relationships between the composition of the AOB and AOA communities and soil properties were estimated using the Mantel test in Mothur. Dendrograms of AOB and AOA community structures were constructed using the Yue & Clayton measure of dissimilarity and unweighted pair group method with arithmetic mean (UPGMA) clustering in Mothur (37).

### Accession number of DNA sequences

Pyrosequencing data were deposited in the DDBJ sequence read archive (DRA) DDBJ databases under accession number DRA002432.

## Results

### Soil properties

Soil properties are shown in Table S1. The soil properties and types and amounts of nitrogen fertilizer applied were different in each farmer’s field. The pH values of samples varied between 4.09 and 7.79, and ten out of thirteen soil samples were classified as acidic (< pH 5.5). The contents of NH_4_^+^-N and NO_3_^−^-N in the soils ranged from 5.0 to 296.8 μg g dry soil^−1^ and 0.2 to 280.2 μg g dry soil^−1^, respectively. According to farmers who were interviewed during the course of this study, eleven sugarcane fields had been receiving 150–200 kgN of nitrogen fertilizer (mainly ammonium sulfate) ha^−1^ year^−1^: field C1 was deserted cultivated land while E7 had been receiving 84 kgN of urea and 450 kg of poultry manure ha^−1^ year^−1^.

### AOB and AOA community size

**Fig. 1:**
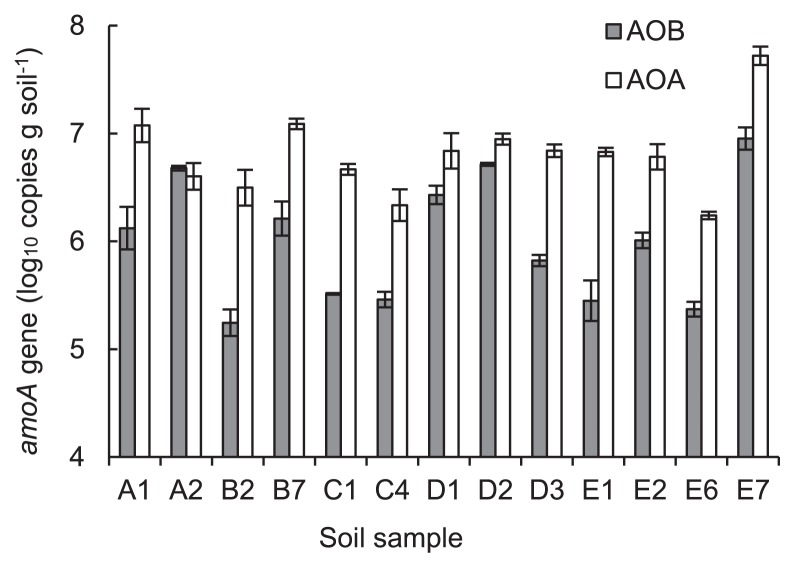
Abundances of AOB and AOA *amoA* genes in soil samples.

The abundances of the AOB and AOA *amoA* genes, which reflected the AOB and AOA populations, were determined by qPCR (Fig. 1). The abundances of AOB *amoA* genes were estimated to be in the range of 1.8 × 10^5^ to 9.2 × 10^6^ gene copies g dry soil^−1^. The E7 soil sample that received poultry manure combined with urea fertilization (Table S1) had the highest abundance of the AOB *amoA* gene. The abundances of the AOA *amoA* genes were estimated to be in the range of 1.7 × 10^6^ to 5.3 × 10^7^ gene copies g dry soil^−1^. The E7 field also had the highest abundance of the AOA *amoA* gene among all the soil samples tested. The abundance of AOB *amoA* was higher than that of AOA in only the A2 soil sample. The ratios of the AOA *amoA* gene to the AOB *amoA* gene ranged from 0.87 to 22.70 among all the soil samples tested.

### Relationship between soil properties and ammonia oxidizer abundance

Relationships were calculated (Table 1) using the values of twelve samples; however, the E7 soil sample was excluded because the pH and PNR of the E7 sample were markedly higher than those of the other samples, which may have led to errors in calculating the relationships. PNR is an important index for evaluating the relationship between the abundance of ammonia oxidizers and nitrification activity. The PNRs of the twelve soil samples were estimated to be in the range of 3.54–12.42 nmole h^−1^ g dry soil^−1^ (Table S1). Based on the correlation analysis, PNR positively correlated with the abundance of the AOB *amoA* gene (r = 0.793, *P* < 0.01) and AOA *amoA* gene (r = 0.648, *P* < 0.05), but did not with the other observed data. The abundance of the AOB *amoA* gene correlated with soil NO_3_^−^-N concentration (r = 0.608, *P* < 0.05). The abundance of the AOA *amoA* gene correlated with water content (r = 0.624, *P* < 0.05). Furthermore, AOB *amoA* gene abundance was associated with AOA *amoA* gene abundance (r = 0.575, *P* = 0.051).

### Community structures of AOB

**Fig. 2:**
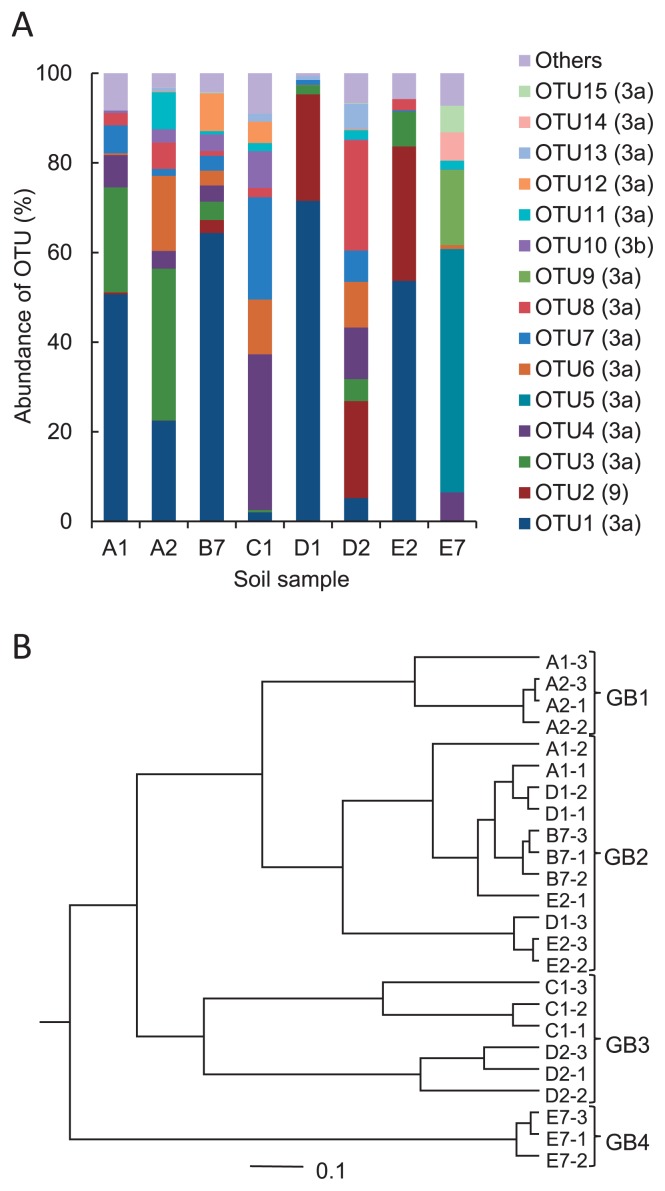
Relative abundance of AOB *amoA* OTUs (A) and the UPGMA cluster tree (B). The relative abundances in panel A show the average of three replicates. The OTU numbers are followed by the *Nitrosospira* cluster numbers, which are given in parentheses. The cluster tree in panel B was constructed using the average of three replicates.

The community structures of AOB were characterized by a 454-pyrosequencing analysis of the AOB *amoA* genes. A total of 61,096 AOB *amoA* gene sequence reads were obtained in triplicate from eight soil samples after excluding chimeric and low-quality sequences (Table S2). The average number of sequence reads in each field was 7,637, and ranged from 5,375 to 11,158. These high quality sequence reads, except for low-abundance OTU (< 0.5% in all samples) sequences, were clustered into 15 OTUs at the 5% cut-off. The representative sequences from dominant OTUs were used in the phylogenetic analysis. We used cluster identification for the AOB *amoA* genes, which was defined previously by Avrahami and Conrad (1). The phylogenetic analysis indicated that all AOB *amoA* OTUs found in the farmed soils were affiliated exclusively with the *Nitrosospira* genus and were placed into clusters 3a, 3b, and 9 (Fig. S2). OTU2 and OTU10 were grouped in cluster 9 and 3b, respectively and the remaining 13 OTUs were grouped in cluster 3a. The average relative OTU abundances from triplicate readings are shown in Fig. 2A and Table S3. Cluster 3a was the most dominant among all soil samples, and cluster 9 (OTU2) was one of the major components in samples D1, D2, and E2, but was minor or undetectable in the other samples. Cluster 3a was a major component in all groups, but its OTU composition differed among the groups. For example, OTU1 was the most abundant and occupied over 50% in A1, A2, B7, D1, and E2 soil samples. However, OTU1 was less abundant in C1 and D2 soil samples and was not detected in the E7 soil sample.

The UPGMA cluster tree constructed using the average of the triplicate data of each sample is shown in Fig. 2B. The community types of AOB in the soils were classified into four different groups consisting of group AOB (GB) 1 (A1 and A2 soil samples), GB2 (A1, B7, D1, and E2), GB3 (C1 and D2), and GB4 (E7) (Fig. 2). The E7 sample of GB4 had been receiving poultry manure and showed the highest pH value of 7.9. The C1 sample of GB3 was deserted arable land and had not been receiving nitrogen fertilizer.

### Community structures of AOA

**Fig. 3:**
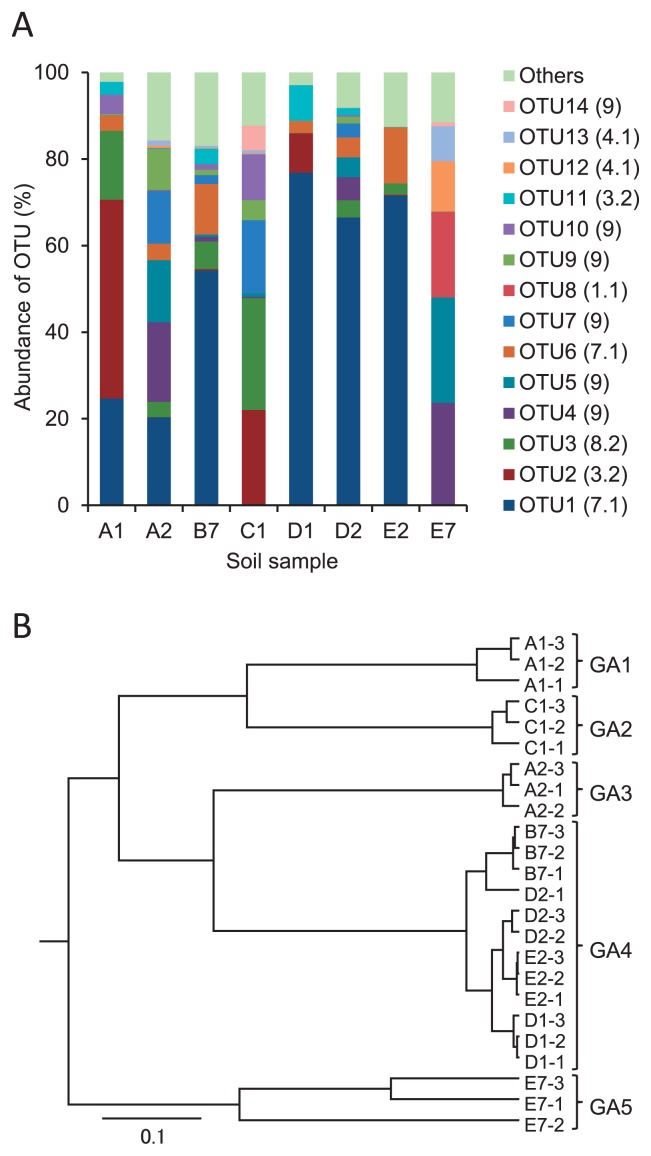
Relative abundance of AOA *amoA* OTUs (A), and the UPGMA cluster tree (B). The relative abundances in panel A show the average of three replicates. The OTU numbers are followed by the *Nitrosophaera* subcluster numbers, which are given in parentheses. The cluster tree in panel B was constructed using the average of three replicates.

A total of 115,492 AOA *amoA* gene sequence reads were obtained from eight soil samples using the same procedure as that for the analysis of AOA. The average number of sequence reads in each field was 14,437 and ranged from 8,609 to 26,022 (Table S2). These high quality sequence reads were clustered into 14 OTUs at the 7% cut-off. The representative sequences from dominant OTUs were used in the phylogenetic analysis. We used cluster and subcluster identification for the AOA *amoA* genes, which was defined by Pester *et al.* (31). The phylogenetic analysis indicated that all AOA *amoA* OTUs found in the farmed soils were affiliated exclusively with the *Nitrososphaera* cluster (Fig. S3). The *Nitrososphaera* cluster included subcluster 1.1 (OTU8), 3.2 (OTU2 and 11), 4.1 (OTU12 and 13), 7.1 (OTU1 and 6), 8.2 (OTU3), and 9 (OTU4, 5, 7, 9, 10, and 14). The average relative OTU abundances from triplicate readings are shown in Fig. 3A and Table S4. Their OTU compositions differed among the individual groups. OTU1 (subcluster 7.1) was the most abundant, occupied over 50% in the B7, D1, D2, and E2 soil samples, and was of secondary dominance in the A1 sample. However, OTU1 was not detected in the C1 or E7 sample, in which the compositions of OTUs were apparently different from those of the other groups. The UPGMA cluster tree constructed using the average of the triplicate data of each sample is shown in Fig. 3B. The community types of AOA in the soils were classified into five groups consisting of group AOA (GA)1 (A1 soil sample), GA2 (C1), GA3 (A2), GA4 (B7, D1, D2, and E2), and GA5 (E7) (Fig. 3B).

### Relationship between environmental factors and AOB and AOA communities

**Fig. 4:**
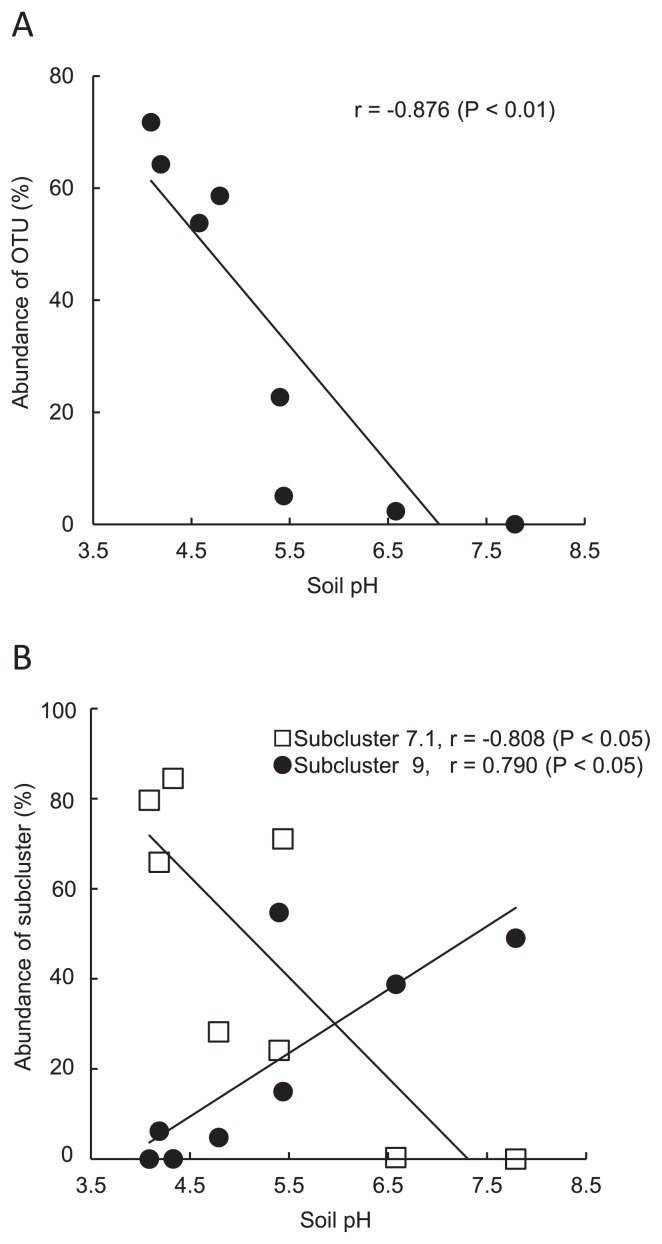
Relationship between the relative abundance of AOB *amoA* OTU1 and soil pH (A), and that between the relative abundances of AOA subclusters (7.1 and 9) and pH (B).

The relative contribution of environmental factors to shaping the community structures of AOB and AOA in agricultural (farmers’) field soils remains unclear. The effects of the six selected environmental variables (pH, NH_4_^+^, NO_3_^−^, available phosphate, and total carbon and nitrogen contents) on the composition of AOB and AOA were evaluated by the Mantel test (Table 2). Soil pH was the most significantly influential, although multiple factors significantly influenced these community structures, suggesting that the community structures of AOB and AOA could be shaped by a complex effect consisting of different environmental factors. A pairwise analysis was performed to evaluate the effects of the individual factors on each OTU’s abundance, and the results obtained showed a negative correlation between the relative abundance of AOB *amoA* OTU1 and soil pH (r = −0.876, *P* < 0.01) (Fig. 4A). This analysis also revealed a negative correlation between the relative abundance of AOA *amoA* subcluster 7.1 (OTU1 and 6) and soil pH (r = −0.808, *P* < 0.05) and a positive correlation between the relative abundance of AOA *amoA* subcluster 9 (OTU4, 5, 7, 9, 10 and 14) and soil pH (r = 0.790, *P* < 0.05) (Fig. 4B). The other environmental factors did not correlate with the OTUs of the AOB and AOA subclusters.

## Discussion

### Abundance of AOB and AOA

The abundance and diversity of AOB and AOA have been investigated using soils from controlled experimental fields conducted for research purposes. Consistent with most of these studies, the abundances of AOA were higher than those of AOB in twelve out of the thirteen sugarcane fields examined in the current study, and the ratios of AOA to AOB were in a range similar to those reported previously for various agricultural soils (10, 14, 19, 24, 28, 38). A number of studies have been conducted to evaluate the relative contribution of AOB and AOA to nitrification in various soils (6). These studies provided evidence that both AOB and AOA were significant players in ammonia oxidation, while the relative importance of AOB and AOA was found to vary in soil depending on environmental conditions (5, 19, 21, 30, 45). We assessed the relationship between the abundances of their *amoA* and PNR. AOB and AOA *amoA* gene abundances correlated with PNR, indicating that both AOB and AOA contributed to ammonia oxidation in the soils from sugarcane fields.

### Community structure of AOB and AOA

The AOB communities consisted exclusively of the genus *Nitrosospira* in the sugarcane field soils. AOB were divided into three genera: *Nitrosomonas* (*β*-proteobacteria), *Nitrosospira* (*β*-proteobacteria), and *Nitrosococcus* (*γ*-proteobacteria) (16); the former two genera are generally found in soil environments that include agricultural fields, forests, and grassland. *Nitrosospira* were previously identified as the dominant genus in many agricultural field soils (22). Consistent with these findings, *Nitrosospira* was the solely dominant genus in the present study, and *Nitrosomonas* was not found in any of the soil samples, despite having deeply sequenced *amoA* by pyrosequencing. *Nitrosomonas* was previously detected in an agricultural soil that had been periodically treated with large amounts of nitrogen fertilizer (11). The amount of nitrogen fertilizer applied to the sugarcane fields examined in this study may not have been sufficient to increase the *Nitrosomonas* population size to a detectable level.

All AOA isolates and environmental clones are placed into the phylum *Thaumarchaeota* in domain Archaea (41). AOA are classified into five clusters consisting of *Nitrosopumilus*, *Nitrososphaera*, *Nitrosocaldus*, *Nitrosotalea*, and *Nitrososphaera* sister clusters (31). Four of these clusters, excluding *Nitrosocaldus*, have been detected in various environments including soils (31). Recent studies showed that *Nitrososphaera* was the most dominant cluster in agricultural soils (20). In the present study, *Nitrososphaera* was the dominant cluster and the AOA communities in the sugarcane field soils were composed exclusively of *Nitrososphaera*.

### Effects of pH on AOB and AOA communities

The community structures of AOB and AOA in soils have been shown to be affected by various environmental factors such as pH (15, 29), nutrient level (18), soil type (27, 28), temperature (12), land use (40), and geography (4). In the present study, the Mantel test revealed that, among the six selected environmental variables examined, soil pH was the most significant factor affecting the community composition of AOB and AOA. A previous study using experimental fields also indicated that soil pH was one of the most important factors for shaping the community structure of both AOB and AOA (7). AOB are thought to grow poorly under acidic conditions due to the low levels of available ammonia (NH_3_ ), which is believed to be the actual substrate for autotrophic ammonia oxidation rather than ammonium (NH_4_^+^) (43). On the other hand, recent studies have shown that AOA has higher affinity than AOB for ammonia and could more easily adapt to environments with low available ammonia concentrations such as those in acidic soils (26, 29). In the acidic soil of tea fields, PNR positively correlated with AOA *amoA* abundance, but not with AOB *amoA*, and the AOA/AOB ratio increased with decreases in soil pH (48). However, in the present study, PNR positively correlated with both AOB and AOA *amoA* abundances, and the ratio of AOA/AOB was not significantly affected by soil pH. These results indicated that the communities of AOB and AOA may change and adapt to each soil pH and that both AOB and AOA can contribute to nitrification regardless of soil pH. The AOB and AOA community structures in the E7 soil sample, which had the highest soil pH, were completely different from those in the B7 and D1 soil samples, which had the lowest pH values. The soil pH of the E7 sample appeared to be increased by the addition of poultry manure, which contains high amounts of Ca. Conversely, the soil pH of the B7 and D1 soil samples had been acidified by the application of ammonium sulfate. Fertilization has direct and indirect effects on AOB and AOA community structures through the supply of ammonium and acidification or neutralization of soil (7, 10).

*Nitrosospira* clusters 2 and 4 have been detected in some acidic and neutral agricultural soils (22), although they were not found in the soils tested in the current study. However, a negative correlation was observed between the relative abundance of AOB *amoA* OTU1 and soil pH (r = 0.876, *P* < 0.01) in these farmed soils. These results suggest that the particular phylogenetic lineages of *Nitrosospira* had been selected by soil pH and fertilizer management. On the other hand, in the present study, the relative abundances of *Nitrososphaera* subcluster 7.1 (AOA *amoA* OTU1 and 6) increased with decreases in soil pH, and negative correlations were observed between them and pH (r = −0.808, *P* < 0.05). *Nitrosotalea devanaterra* was recently isolated from one acidic soil and characterized as an obligate acidophile (23). The *Nitrosotalea* subcluster was identified as an abundant lineage in several acidic agricultural soils (31). However, the *Nitrosotalea* subcluster was not found in any of the soil samples tested in the current study, despite several soils having low pH values ranging from 4.17 to 4.79. These results suggest that some *Nitrososphaera* subcluster 7.1-related communities may adapt to acidic conditions and become the dominant group in the acidic soils of sugarcane fields. Conversely, a positive correlation (r = 0.790, *P* < 0.05) was found between the relative abundance of *Nitrososphaera* subcluster 9 and soil pH. This subcluster appeared to be acid-sensitive and play a major role in nitrification in the neutral and alkaline soils. Hu *et al.* (17) demonstrated that the individual lineages of AOB and AOA in Chinese soils significantly changed along with the soil pH gradient. Nicol *et al.* (29) indicated that the composition of the phylotypes of AOB and AOA changed in long-term experimental field soils with different pHs, and suggested that AOB and AOA had distinct physiological characteristics and ecological niches.

## Conclusion

By pyrosequencing the *amoA* gene from AOB and AOA, found in agricultural (farmers’) sugarcane field soils, the present study showed distinct relationships between soil properties and AOB and AOA community structures, suggesting that niche differentiation between the cluster and subcluster levels of AOB and AOA depended on the environmental conditions of the soil. Soil pH was the most important factor shaping the community structures of AOB and AOA, and OTU1 of the *Nitrosospira* cluster 3a subcluster and *Nitrosophaerae* subcluster 7.1 adapted to and dominated the acidic soil. These results provide fundamental data for developing nitrification control technologies, such as nitrification inhibitors, which require information for targeting AOB and AOA for their efficient development.

## Supplementary Information



## Figures and Tables

**Table 1:** Pearson correlations (*r*) between soil properties, potential nitrification rates (PNR), and abundances of AOB and AOA *amoA* genes

	Water content	pH	TN	TC	NO_3_^−^-N	AOA abundance	PNR
AOB abundance	0.549†	NS	NS	NS	0.608*	0.575†	0.793**
AOA abundance	0.624*	NS	NS	NS	0.574†	—	0.648*
PNR	NS	NS	NS	NS	NS	—	—

** *P* < 0.01;

* *P* < 0.05;

† *P* < 0.1;

NS, not significant at *P* ≥ 0.1

**Table 2:** Mantel correlations (*r_M_*) between AOB and AOA community structures and soil characteristics

	*r_M_*	
	
	AOB structure	AOA structure
pH	0.750***	0.637***
TN	0.318***	0.189*
TC	0.488***	0.466**
NH_4_^+^-N	−0.042	0.209*
NO_3_^−^-N	0.134*	0.064
Available-P	0.336***	0.337***

*** *P* < 0.001,

** *P* < 0.01,

* *P* < 0.05
