# Supplementary material for: Environmental Factors Shaping the Community Structure of Ammonia-Oxidizing Bacteria and Archaea in Sugarcane Field Soil
Source: Microbes Environ. 2014 Dec 27;30(1):21–8. doi: 10.1264/jsme2.ME14137 (PMC4356460; doi:10.1264/jsme2.ME14137)
Supplement: Supplementary file 1 [file 30_21_s1.pdf]

Table S1 Soil properties, potential nitrification rate (PNR) and fertilization[a]

Soil sample	Water[b] (mg g^{-1})	pH	TN (mg g^{-1})	TC (mg g^{-1})	NH_4^+-N (μg g $soil^{-1}$)	NO_3^--N (μg g $soil^{-1}$)	Available-P (μg g $soil^{-1}$)	PNR[c]	Fertilizer[d] kgN ha^{-1} $year^{-1}$
A1	20.5	4.79	0.18	1.32	127.1	86.8	99.6	10.40	208[e]
A2	23.1	5.40	0.20	1.40	29.8	81.4	91.4	11.66	180[e]
B2	20.3	4.33	0.21	1.42	296.8	35.4	89.8	3.54	256[e]
B7	26.2	4.19	0.22	1.75	9.8	266.2	81.1	11.12	256[e]
C1	19.1	6.58	0.16	1.29	5.0	0.2	63.3	9.74	0[f]
C4	22.1	5.23	0.17	1.31	15.8	2.1	60.6	4.32	192[e]
D1	25.5	4.09	0.19	1.42	13.2	280.2	88.4	7.99	288[e]
D2	23.5	5.44	0.16	1.28	28.6	84.4	83.7	12.42	216[e]
D3	26.6	4.56	0.22	2.04	6.4	43.7	79.8	8.73	192[g]
E1	22.1	4.58	0.16	1.27	13.8	19.2	110.6	5.40	158[g]
E2	21.3	4.58	0.17	1.15	43.4	55.8	112.6	5.20	216[e]
E6	14.7	6.32	0.20	1.73	32.9	6.4	63.0	4.72	256[e]
E7	26.7	7.79	0.22	3.71	3.3	41.5	107.8	83.92	102[h]

[a] All values barring those of PNR and fertilizer were excerpted from a report by Tago *et al.*(doi:10.1264/jsme2.ME14124); [b]water content; [c]potential nitrification rate (nmole nitrite production/hr/g dry soil); [d]based on interviews with farmers; [e]received ammonium sulfate; [f]deserted cultivated land; [g]received slow-release nitrogen fertilizer; [h]received urea and poultry manure.

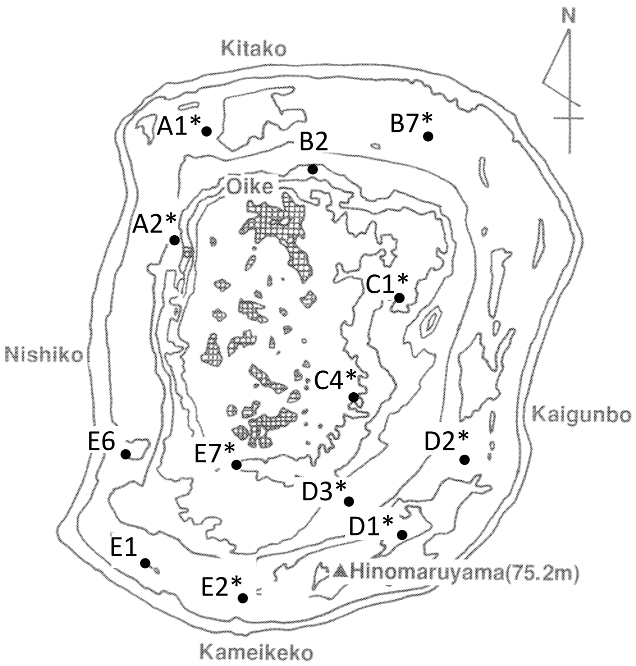

Fig. S1 Map of sampling fields. Field numbers are indicated. The sampling fields used for pyrosequencing analysis are represented by asterisks. A contour map is provided by the Geospatial Information Authority of Japan.

Table S2 Number of sequence reads of AOB and AOA *amoA* genes.

Soil sample	AOB		AOA	
	Sequence reads/field	Sequence reads/subsample	Sequence reads/field	Sequence reads/subsample
A1	5,375	2,818	11,254	2,706
		1,753		4,765
		804		3,783
A2	11,158	3,373	14,716	3,828
		4,160		6,654
		3,625		4,234
B7	8,695	2,814	15,703	5,848
		3,080		4,898
		2,801		4,957
C1	7,823	2,376	17,212	3,733
		2,314		6,501
		3,133		6,978
D1	5,593	1,815	10,746	3,755
		1,962		4,269
		1,816		2,722
D2	7,168	2,805	11,230	4,123
		1,572		4,480
		2,791		2,627
E2	6,013	2,060	8,609	2,713
		1,301		3,493
		2,652		2,403
E7	9,271	2,892	26,022	11,093
		3,290		10,207
		3,089		4,722
	61,096		115,492	

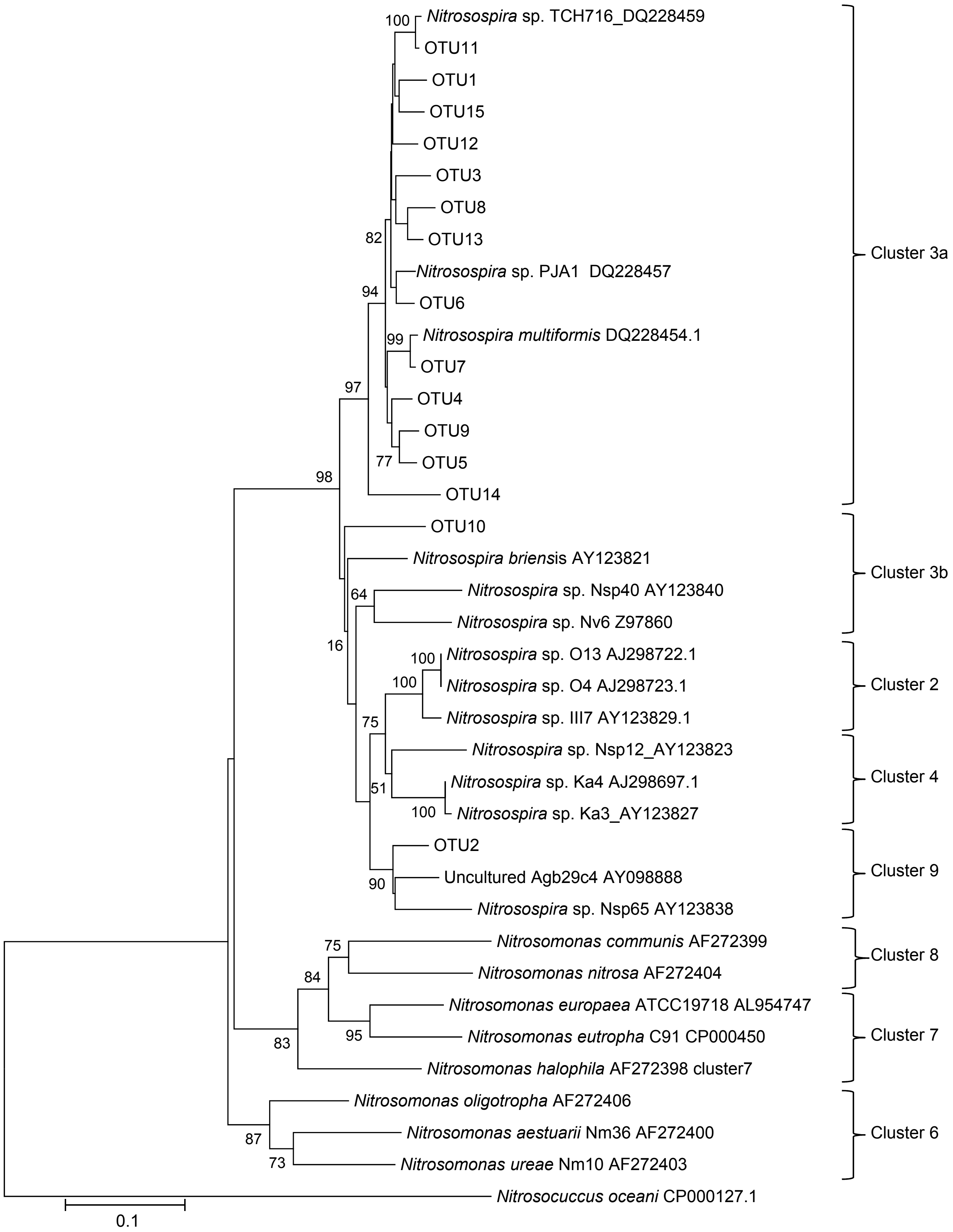

Fig. S2 Phylogenetic tree of AOB *amoA* OTUs. Accession numbers of reference sequences in the NCBI database are indicated. The *amoA* gene sequence of *Nitrosococcus oceanii* was used as an outgroup. Branch nodes with >50% supporting bootstrap values (1000 replicates) are shown.

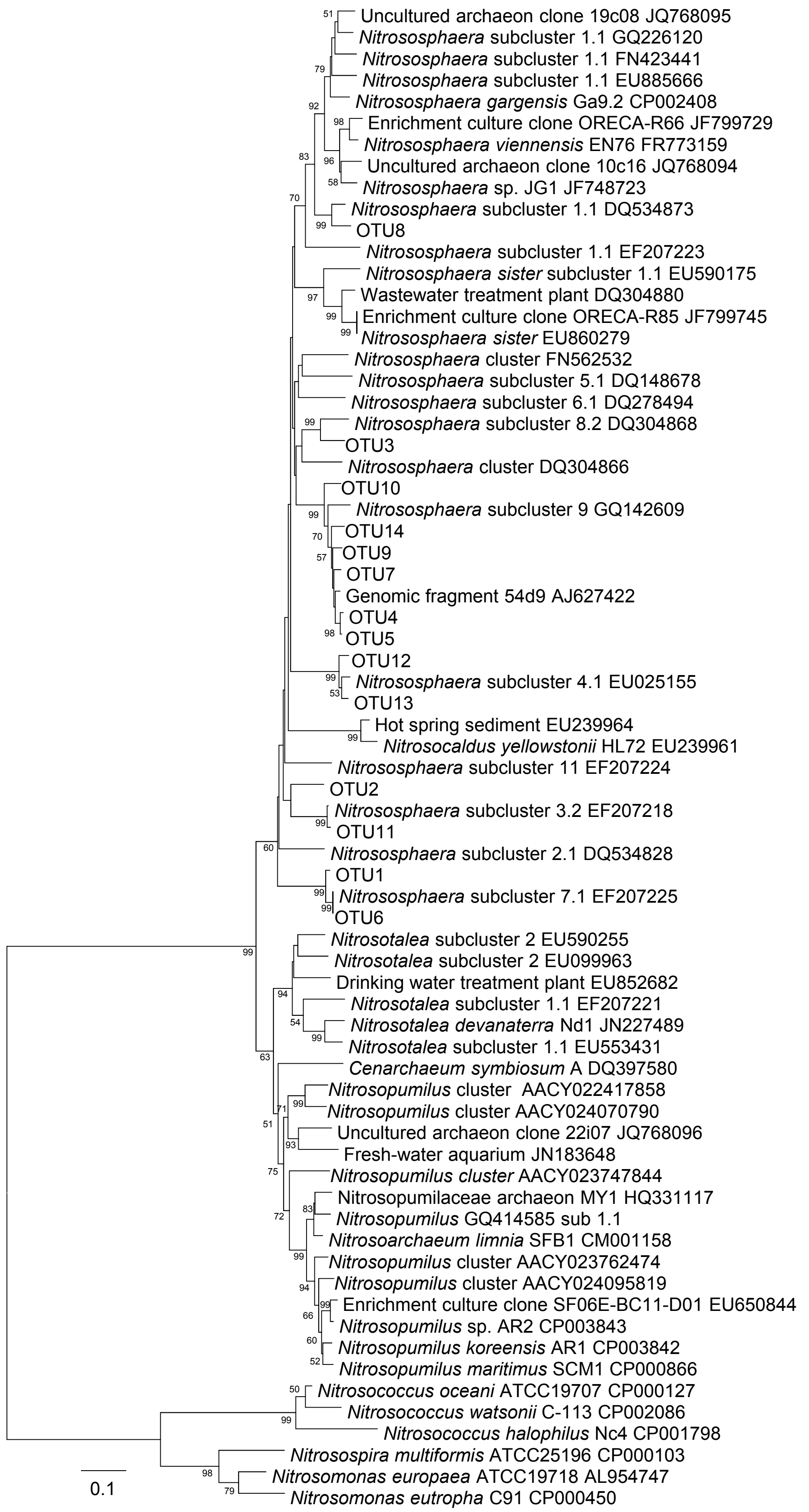

Fig. S3 Phylogenetic tree of AOA *amoA* OTUs. Accession numbers of reference sequences in the NCBI database are indicated. The *amoA* gene sequences of bacterial AOB was used as an outgroup. Branch nodes with >50% supporting bootstrap values (1000 replicates) are shown.

Table S3 *Nitrosospira* cluster composition in the soils.

Subcluster	OTU No.	Field No.							
		A1	A2	B7	C1	D1	D2	E2	E7
		Relative abundance (%)							
3a	1, 3-9, 11-15	90.7	93.8	89.1	82.8	75.6	71.5	64.2	92.6
3b	10	0.6	2.9	3.7	8.2	0.0	0.2	0.0	0.1
9	2	0.4	0.0	2.9	0.0	23.8	21.7	30.0	0.0

Table S4 *Nitrosophaera* subcluster composition in the soils.

Subcluster	OTU No.	Field No.							
		A1	A2	B7	C1	D1	D2	B2	E7
		Relative abundance (%)							
7.1	1,6	28.2	24.1	65.9	0.3	79.7	71.1	84.5	0.0
9	4,5,7,9,10,14	4.8	54.8	6.2	38.8	0.0	15.0	0.0	49.1
3.2	2,11	48.9	0.1	3.9	21.8	17.3	1.7	0.3	0.0
8.2	3	15.9	3.6	6.3	25.9	0.1	4.0	2.6	0.0
4.1	12,13	0.0	1.7	0.7	0.9	0.0	0.1	0.0	19.6
1.1	8	0.0	0.1	0.0	0.0	0.0	0.0	0.0	19.8
